# Metabolite Profiling of Diverse Rice Germplasm and Identification of Conserved Metabolic Markers of Rice Roots in Response to Long-Term Mild Salinity Stress

**DOI:** 10.3390/ijms160921959

**Published:** 2015-09-11

**Authors:** Myung Hee Nam, Eunjung Bang, Taek Yun Kwon, Yuran Kim, Eun Hee Kim, Kyungwon Cho, Woong June Park, Beom-Gi Kim, In Sun Yoon

**Affiliations:** 1 Environmental Risk and Welfare Research Team, Korea Basic Science Institute, Seoul 02855, Korea; E-Mails: nammh@kbsi.re.kr (M.H.N.); uran115@naver.com (Y.K.); kw.cho253@gmail.com (K.C.); 2 Omics System Research Team, Korea Basic Science Institute, Seoul 03759, Korea; E-Mail: bej@kbsi.re.kr; 3 Molecular Breeding Division, National Academy of Agricultural Science, Jeonju 565-851, Korea; E-Mails: trkwon@korea.kr (T.Y.K.); bgkimpeace@korea.kr (B.-G.K.); 4 Protein Structure Team, Korea Basic Science Institute, Cheongju 28119, Korea; E-Mail: keh@kbsi.re.kr; 5 Department of Molecular Biology, Institute of Nanosensor and Biotechnology, Dankook University, Yongin-si, Gyeonggi-do 448-701, Korea; E-Mail: parkwj@dankook.ac.kr

**Keywords:** rice germplasm, salt stress, root, ^1^H-NMR, metabolite markers

## Abstract

The sensitivity of rice to salt stress greatly depends on growth stages, organ types and cultivars. Especially, the roots of young rice seedlings are highly salt-sensitive organs that limit plant growth, even under mild soil salinity conditions. In an attempt to identify metabolic markers of rice roots responding to salt stress, metabolite profiling was performed by ^1^H-NMR spectroscopy in 38 rice genotypes that varied in biomass accumulation under long-term mild salinity condition. Multivariate statistical analysis showed separation of the control and salt-treated rice roots and rice genotypes with differential growth potential. By quantitative analyses of ^1^H-NMR data, five conserved salt-responsive metabolic markers of rice roots were identified. Sucrose, allantoin and glutamate accumulated by salt stress, whereas the levels of glutamine and alanine decreased. A positive correlation of metabolite changes with growth potential and salt tolerance of rice genotypes was observed for allantoin and glutamine. Adjustment of nitrogen metabolism in rice roots is likely to be closely related to maintain the growth potential and increase the stress tolerance of rice.

## 1. Introduction

Salinity is a major environmental constraint to crop productivity worldwide. Rice is one of the most salt-sensitive crops, and the grain yield is very susceptible to soil salinity in both arid and semi-arid regions. It has been known that the sensitivity of rice to salt stress greatly depends on growth stages, organ types and cultivars [1,2,3,4]. For example, rice is more sensitive to salinity during early seedling growth and flowering than other growth stages [5]. Younger seedlings are more susceptible to salinity than older seedlings, and root growth is more susceptible than shoot growth [2,3]. The half maximum inhibitory concentration (IC_50_) of root growth of young rice seedlings is less than 43 mM NaCl [6]. Therefore, the ability of seedling growth under salt stress conditions is an indicator of the salt tolerance of rice. There is a positive correlation between salt resistance at the seedling stage and grain yield in saline soil. Salt tolerance of a plant is a very complicated process that involves numerous physiological and molecular networks. As in other plants, the salt tolerance of rice primarily depends on the ion homeostasis. During the initial phase of salt stress, diverse mechanisms, such as secretion, vacuolar sequestration and root-shoot transport of salt, are involved in keeping the cellular Na^+^ and Cl^−^ ions at a nontoxic level. Among rice cultivars and individual plants, a dramatic variability in the sodium and chloride level was observed, and the concentration of these ions within a plant negatively correlated with the survival period under saline conditions [2]. The second phase of growth inhibition by salt stress largely depends on the metabolic interference by cytosolic sodium and chloride ions. They interfere with the membrane transport activity, change the K^+^ ion balance and membrane potential, limit water availability and inhibit many enzyme activities that lead to sequential and detrimental metabolic changes toward plant death [7,8]. Therefore, it is expected that numerous physiological and molecular components participate in the plant salt tolerance, probably with differential roles depending on tissues, developmental stages, genotypes and plant species. The salt overly-sensitive (SOS) pathway or high affinity potassium (HKT) transporter is one of the conserved molecular components of the salt tolerance mechanism to maintain ion homeostasis in many plant species, including rice [9,10]. So far, previous reports indicated that regulation of osmolyte biosynthesis, sodium efflux and/or compartmentation and the ROS scavenging system have effects on enhancing the salt tolerance of rice. It also has been reported that salt tolerance of rice can be increased by the regulation of transcription factors and signaling components involved in ABA signaling, such as pyrabactin resistance-like (PYL)-type ABA receptors, SNF1-related protein kinase 2 (SnRK2) or bZIP transcription factors [11,12,13]. However, the adoption of these salt-responsive strategies may be significantly different in tolerant and sensitive cultivars, both at the cellular and whole tissue levels.

The newly-developed high throughput technology of functional genomics and omics studies provides the comprehensive network of plant salt tolerance. Metabolite profiling is a valuable tool for the investigation of plant responses to the environment at the molecular level. The comprehensive, quantitative and qualitative measurement of cellular metabolites prepared from cells or tissues exposed to stress conditions can provide a broad overview of the biological responses of plants to stresses [14,15]. Of the analytical platforms used in metabolomics, nuclear magnetic resonance (NMR) spectroscopy is a very useful tool in the study of plant stress responses, because it can simultaneously detect diverse groups of metabolites, and the spectrum signal is highly reproducible and proportional to their amount, making it possible to directly compare concentrations of metabolites [16].

The rice germplasms with differential salt sensitivity are good materials to identify molecular components contributing to salt tolerance. In the present study, we aimed to exploit conserved and differential metabolic changes in rice roots in response to long-term salt stress among different rice genotypes. To identify salt-responsive metabolic markers conserved in rice genotypes, 38 rice accessions were subjected to mild salt stress for two weeks, and their metabolites from roots were profiled by ^1^H-NMR spectroscopy. Our data identified five conserved salt responsive metabolic markers of rice roots, *i.e*., sucrose, allantoin, glutamate, glutamine and alanine. A positive correlation of metabolite changes with growth potential and salt tolerance of rice genotypes was observed for allantoin and glutamine, suggesting that the adjustment of nitrogen metabolism in rice roots might be closely related to maintain growth potential and increase the stress tolerance of rice.

## 2. Results

### 2.1. Growth Potential of 38 Rice Genotypes under Long-Term Mild Salinity Stress

**Figure 1:**
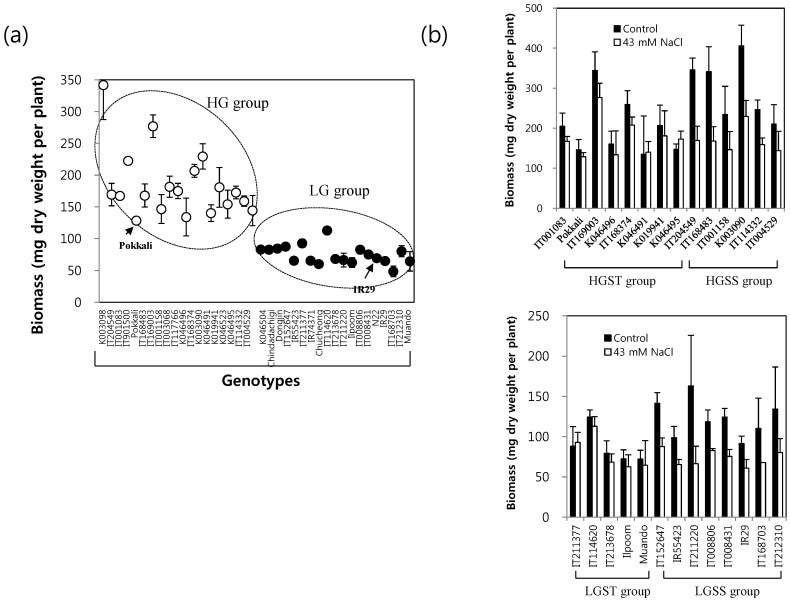
(**a**) Rice germplasms differing in the ability of biomass accumulation in the presence of moderate salinity. Imposition of 43 mM NaCl supplemented to half strength Yoshida’s nutrient solution started on rice seedlings of the three or four foliar leaf stage, which is known as a sensitive growth stage to salinity and ended at 14 days after starting the salinity imposition. Biomass was determined after the end of salinity treatment. Reference rice genotypes were used for cv. “Pokkali” (129 ± 5.2 mg) as a relatively tolerant genotype and for cv. “IR 29” (65 ± 5.5 mg) as a relatively sensitive one. Each mark represents the mean of nine plants. HG, high growth group; LG, low growth group; (**b**) Changes in the biomass accumulation of rice genotypes in response to 43 mM NaCl. Genotypes showing less than a 20% inhibition of biomass accumulation by salt stress were grouped as the salt-tolerant (ST) and genotypes with more than 30% inhibition as the salt-sensitive (SS) group. Bars indicate SD.

Rice germplasms showing differential growth responses under a given stress condition can be good sources to investigate stress tolerant mechanism. In the present study, a subset of rice germplasms was obtained from the core collection of the National Agrobiodiversity Center of Rural Development Administration (Korea), and their growth responses to mild salinity stress were determined by biomass measurements in a hydroponic culture system. Figure 1a shows the ability of the biomass accumulation of the 38 rice genotypes at the end of the 43 mM NaCl treatment for 14 days. There was a considerable genotypic variation of growth potential, and we categorized the 38 rice genotypes into two subgroups, *i.e*., the relatively high growth (HG; 19 accessions) and low growth (LG; 19 accessions) phenotype groups, respectively. HG denotes genotypes showing biomass accumulation of more than 125 mg dry weight/plant in the presence or absence of salt stress, respectively. We then grouped the 38 rice genotypes into 13 salt-tolerant and 14 salt-sensitive rice accessions based on the degree of salt inhibition of the biomass accumulation (Figure 1b). Genotypes showing less than 20% inhibition of biomass accumulation by salt stress were grouped as salt-tolerant (ST) and genotypes with more than 30% salt inhibition as salt-sensitive (SS) group. According to our grouping, eight high growth and salt-tolerant (HGST) genotypes and eight low growth and salt-sensitive (LGSS) genotypes were separated. In our experimental system, the growth of the salt tolerant reference cv. “Pokkali” was decreased 12% by long-term mild salinity, whereas 34% growth reduction was observed in the salt-sensitive cv. “IR29”. Our data indicate that high growth potential under mild salinity stress is correlated with salt tolerance for some genotypes, while others are not, implicating that the mechanism of salt tolerance might be differential among rice genotypes.

### 2.2. ^1^H-NMR Spectroscopy of Root Metabolites of 38 Rice Genotypes and Multivariate Statistical Analyses Relating to the Growth Rate and Salt Response of Rice Roots

**Figure 2:**
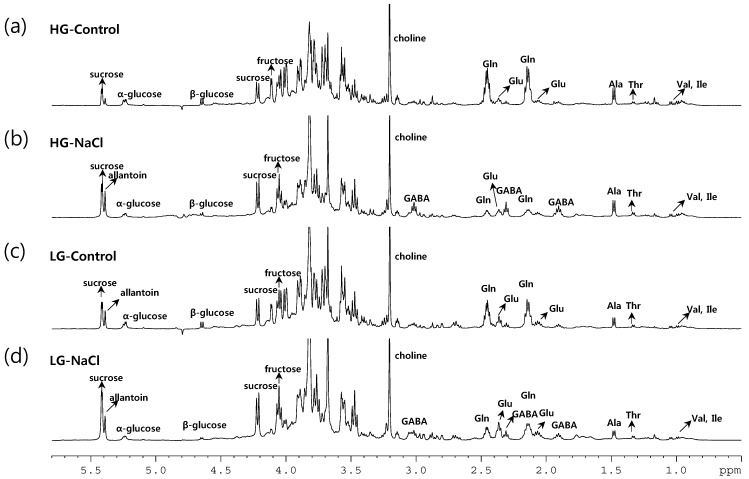
Typical 500-MHz ^1^H-NMR spectra of the aqueous extract from the rice roots of the HG control (**a**); HG salt treated (**b**); LG control (**c**); and LG salt treated (**d**). Gln, glutamine; Glu, glutamate; Ala, alanine; Thr, threonine; Val, valine; Ile, isoleucine; GABA, γ-aminobutyric acid.

In an attempt to exploit stress markers in rice roots in relation to salt tolerance, the metabolic content of the roots of 38 genotypes grown under control and salt stress conditions were analyzed with ^1^H-NMR spectroscopy. Figure 2 shows representative ^1^H-NMR spectra of root extracts obtained from high growth (HG, a and b) and low growth group (LG, c and d) in the presence (b and d) or absence (a and c) of mild salt stress, respectively. Our ^1^H-NMR spectroscopy detected diverse metabolites for sugar and nitrogen metabolism, such as sucrose, fructose, glucose, amino acids and derivatives. The relative peak intensities of significant salt-responsive metabolites were generated from the normalized binning data of ^1^H-NMR spectra.

**Figure 3:**
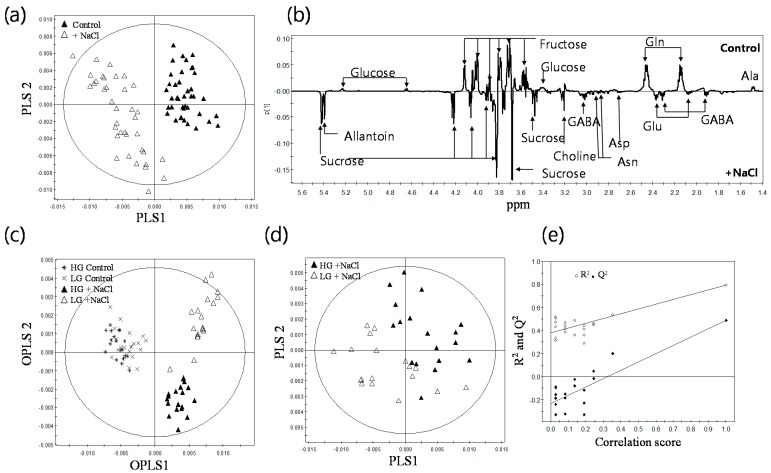
Multivariate statistical analysis of ^1^H-NMR data depending on the growth type and salt response of rice roots. (**a**) PLS-DA score plot from total of 76 samples obtained from the control and 38 mild salt-treated genotypes; (**b**) PLS-DA loading plot of the control and salt-treated groups; (**c**) OPLS/O2PLS-DA score plot of four groups: HG control, LG control, HG + salt, LG + salt; (**d**) PLS-DA score plot to distinguish the differences between the salt-treated HG and LG groups; (**e**) The validation plot of permutation test for the PLS-DA of (**d**). The R^2^Y intercept is at 0.381 and the Q^2^Y intercept is at −0.218. The circles in the OPLS/O2PLS-DA and PLS-DA score plots indicate the tolerance ellipse based on Hotelling’s T^2^. The *X*-axis denotes correlation scores between original and permuted data.

Multivariate data analysis is used to reduce the dimensionality of the multivariate dataset and to identify the differences or similarities among the samples. Each point of the score plot in multivariate analysis represents an individual sample. Grouping and outliers of samples among the samples can be easily observed in a score plot. PLS-DA, a supervised multivariate data analysis method, was performed to investigate intrinsic variation in ^1^H-NMR data obtained from 38 independent genotypes of rice with or without mild salt treatment. The PLS-DA score plot of ^1^H-NMR spectra showed a clear separation between the control and salt-treated groups (Figure 3a). The goodness of fit and predictability were qualified by R^2^Y and Q^2^Y, respectively, where zero was no variation explained, and one, for which 100% variation was accounted [17,18]. The R^2^Y shows the amount of Y variables explained by the model after cross validation and gives an overview about the fitting of the model, while Q^2^Y gives information about the predictive quality of the model. For the PLS-DA, the R^2^Y value of 0.93 and Q^2^Yvalue of 0.91 were higher in the permutation test than the real model, suggesting good predictability and goodness of fit.

The PLS-DA loading plot was generated to visualize salt-responsive metabolites (Figure 3b). The loading plot is preferably sorted by size in order to separate up- and down-regulated spectral regions. Each point of the PLS-DA loading plot represented the most influential spectral region in the calculation of the PLS-DA scores. Therefore, the loading plot can be used to detect metabolites responsible for separation in the data. As shown in Figure 3b, the roots of salt-exposed rice were characterized as having a lower level of glutamine, glucose, alanine and fructose and a higher level of sucrose, allantoin, glutamate and GABA. Next, OPLS/O2PLS-DA was applied to minimize possible contributions from intergroup variability and to further improve the separation of the four groups, including mild salt-treated HG and LG and their corresponding controls. OPLS/O2PLS-DA showed clear separation between the control and salt-treated group (Figure 3c). It also showed a separation between the HG and LG groups obtained from the salt-treated roots of rice (R^2^X = 0.92, R^2^Y = 0.77, Q^2^Y = 0.62), suggesting that the HG and LG groups differentiate salt responses with different metabolic constituents. To discriminate the salt response of the HG and LG groups, we further performed PLS-DA using the salt treated-HG and LG groups (Figure 3d). PLS-DA showed a discrimination between these two groups (Figure 3d). Both R^2^Y (0.795) and Q^2^Y (0.49) were higher in the permutation test than in the real model (Figure 3e), suggesting that although there were diverse genetic backgrounds, the inter- and intra-groups of HG and LG, it was possible to discriminate metabolic response by salt between the HG and LG groups.

### 2.3. Metabolic Profiling of 38 Rice Genotypes Revealed Conserved Salt Stress Metabolite Markers of Rice Roots

**Figure 4:**
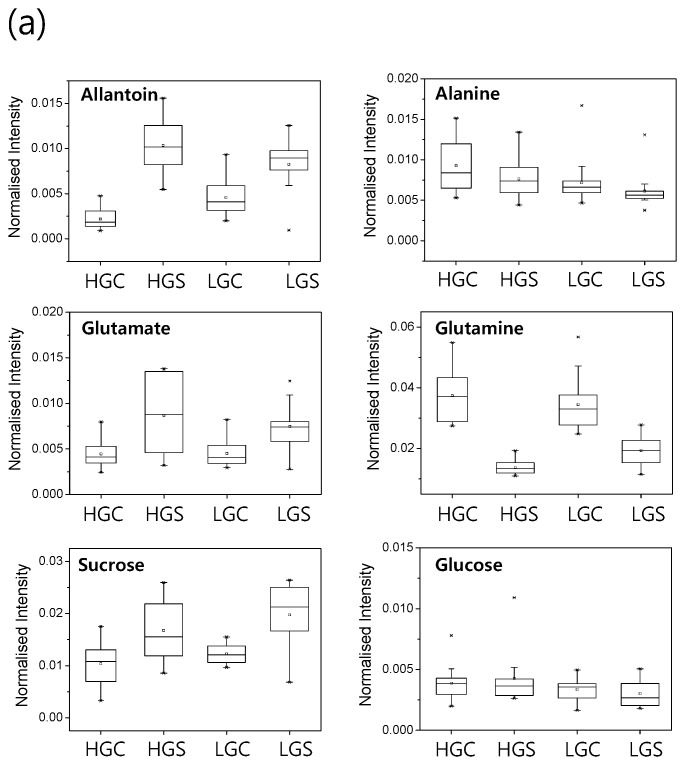

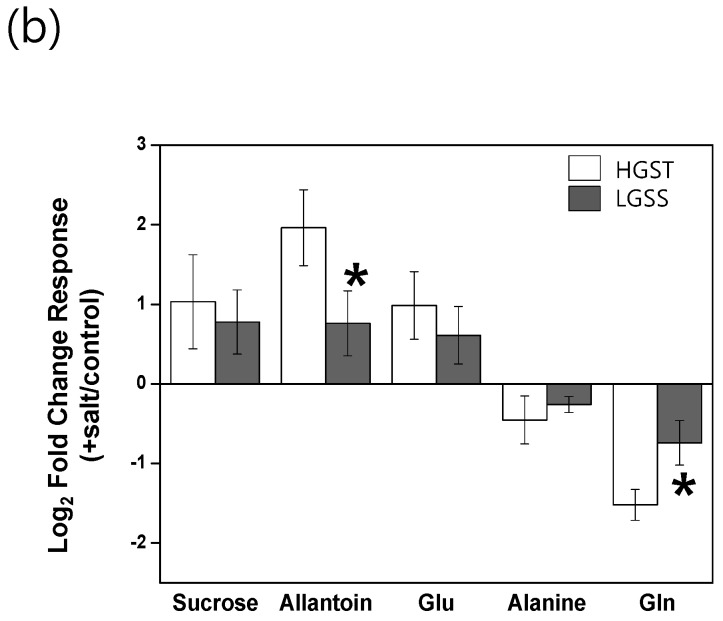
(**a**) Box and whisker plots for the changes of the salt-responsive metabolites in 38 rice genotypes. Maximum and minimum values of a metabolite among each group represented at the upper and lower end of the whisker, respectively, and their 75th and 25th percentiles are represent at the upper and lower end of the box, respectively; (**b**) Comparison of salt-induced fold changes of metabolites between high growth and salt-tolerant (HGST) and low growth and salt-sensitive (LGSS) rice cultivars. Columns indicate the log_2_ of the peak area ratio for metabolites in the control *versus* salt-treated rice roots. The log_2_ of the peak area means the log_2_ ratio of the average peak intensity in the salt-treated rice roots to that in the control roots. A single asterisk indicates a statistically-significant difference (*p* < 0.05). Bars indicate the standard deviation.

From our normalized binning data of ^1^H-NMR spectra, the relative peak intensities of eight distinctive salt-responsive metabolites were quantified and compared among 38 rice genotypes. These metabolites were sugars (sucrose and glucose), amino acids (glutamate, glutamine, alanine and threonine), ureide (allantoin) and choline. Our quantitative analysis indicated that sucrose, allantoin, glutamate and threonine were accumulated by salt stress, whereas the levels of glutamine and alanine were decreased (*p* < 0.05) in roots of most genotypes (Figure S1). Therefore, these metabolites are likely to be salt stress markers that may be conserved in most of the genotypes, and they may be closely related to the salt stress and/or adaptation response of rice roots. Glutamate, glutamine and allantoin are key metabolites for nitrogen assimilation and redistribution. It is noted that rice genotypes with high growth potential (HG group) showed greater changes in these metabolite levels by salt stress than genotypes of low growth potential (LG group) (Figure 4a). If we compared the salt-induced fold changes of these metabolites between high growth and salt-tolerant (HGST group) and low growth and salt-sensitive (LGSS group) rice genotypes, a significant difference (*p* < 0.05) appeared for glutamine and allantoin between the HGST and LGSS group (Figure 4b). These data suggest that the ability to adjust the level of these nitrogen metabolites in rice roots is closely related to maintaining the growth potential and may increase the stress tolerance of rice.

## 3. Discussion

### 3.1. The Glutamate/Glutamine Pathway Plays an Important Role in Rice Roots Exposed to Long-Term Mild Salt Stress

The interaction of salt stress and nitrogen metabolism may involve very complex and multiple networks. The effects of salt stress on the metabolite changes and gene expression related to nitrogen metabolism depend on tissues, organs, developmental stages and plant species [19,20]. However, the causal relationship among salt stress, nitrogen metabolism and the growth response of rice is largely unknown yet. Rice roots preferentially use ammonium as a nitrogen source, and the glutamate/glutamine pathway is a key step of nitrogen assimilation [21]. Metabolite profiling of the rice mutant lacking glutamine synthetase (*OsGS1;*1) revealed that the ammonium assimilation pathway plays a crucial role in coordinating the global metabolic network in rice roots [22]. From our data, the consistent decrease of the glutamine level in all 38 rice genotypes exposed to long-term salt stress emphasized an important role of this metabolite in rice roots (Figure 4a). In contrast to glutamine, the glutamate level was increased by salt stress by more than 1.5-fold in roots of 24 rice genotypes (Table S2). Therefore, the depletion of glutamine in rice roots by long-term salinity stress is likely to be correlated, at least in part, to the increment of glutamate. Siahpoosh *et al*. (2012) [23] reported metabolic depletion syndrome in roots of salt-sensitive rice cultivars exposed to long-term high salt stress (100 mM NaCl) for more than 30 primary metabolites of sugars and amino acids, including glutamine and glutamate. They suggested that this metabolic depletion may be a key factor to limit the growth of young vegetative rice under the prolonged salt stress condition. In our metabolite profiling of 38 rice genotypes under the long-term mild salt stress (43 mM NaCl), however, such a global metabolic depletion was not observed, even in salt-sensitive genotypes. This discrepancy may be due to the difference of the severity of salt stress and the experimental condition. From our data, glutamine is the only metabolite that shows depletion by salt stress common to all rice genotypes, implying that glutamine depletion is a very sensitive parameter of salt stress in rice roots.

It is noteworthy that the salt stress-induced fold reduction of the glutamine level in high growth and salt-tolerant groups (HGST) is significantly higher (*p* < 0.05) than in low growth and salt-sensitive groups (LGSS) (Figure 4b). Previous reports indicate that modulation of the glutamine level and nitrogen metabolism by genetic engineering of glutamine synthetase significantly affects the growth phenotype of rice seedlings. For example, a rice mutant lacking *OsGS1;1* showed a growth retardation phenotype under an ammonium accumulation condition [22]. Transgenic rice over-expressing *OsGS1;2* was much more susceptible to drought and salt stress [24]. Hirano *et al*. (2008) [25] suggested that, for rice seedlings, an assimilatory product of ammonium may serve as an endogenous indicator of the nitrogen status involved in the inhibition of seminal root elongation. Taken together, the glutamate/glutamine ratio is likely to be a metabolic indicator of rice roots under the long-term salt stress and plays an important role in long-term salt adaptation responses.

### 3.2. Sucrose Accumulation Is a Conserved Metabolic Response in Rice Roots Exposed to Long-Term Mild Salinity

The levels of soluble sugars are highly sensitive to environmental stresses, and this response may vary with genotypes and stress factors [26]. However, information on the changes in the sugar content during abiotic stress response is very limited. Sanchez *et al*. (2008) [27] suggested that the salt-dependent accumulation of sucrose might be a conserved metabolic response to salinity in plants. Kim *et al*. (2007) [15] reported co-induction of the metabolites involved in glycolysis and sucrose metabolism in *Arabidopsis thaliana* cell cultures for a long-term period (72 h) of salt stress. Sugar accumulation would be a stress-tolerant mechanism, as these sugars serve as osmolytes to prevent dehydration and provide an energy source. In rice suspension cells, a discrete tendency was observed that sugars and sugar alcohols are increased by salt stress [28]. In our metabolite profiling analysis, sucrose accumulation by salt stress was observed in the roots of 29 rice cultivars, suggesting that sucrose accumulation is a common salt stress responses in rice roots (Figure 4a and Figure S1). The source of sucrose accumulation in rice roots is unknown at present. In contrast to sucrose, the glucose content was not significantly changed in the stressed rice roots (Figure 4a and Figure S1), indicating that glucose is not a source of an elevated level of sucrose. Sucrose accumulated in rice roots could be derived from source-to-sink phloem transport, but little is known about the effects of salt stress on sucrose translocation into the phloem [29]. A low sucrose level in rice roots might be a limiting factor for maintaining a high growth potential under salt stress conditions that makes rice plant salt susceptible. In agreement with this, Siahpoosh *et al.* [23] reported a stronger reduction of sucrose by high salt stress in roots of salt-sensitive rice cultivars compared to salt-moderate or salt-tolerant cultivars. They suggest that the carbon demand of root organ is not balanced by the carbon accumulation in rice leaf under high stress condition. Modification of sugar allocation may contribute to the success of the salt acclimation of rice plants. Photosynthetic activity could be an important factor for sugar allocation under stress conditions, and salinity is known to inhibit photosynthesis in a number of plant species. For example, photosynthetic performance and starch metabolism of IR29 (salt sensitive) was significantly reduced compared to that of Pokkali (salt tolerant) rice under salt stress conditions [30], showing that tolerant rice cultivars maintained a relatively higher photosynthetic function after a brief period of acclimation following exposure to salt stress. Kanemura *et al*. (2007) [31] reported a large genotypic variation of the photosynthetic rate among rice germplasm. It will be interesting to investigate the causal relationship between photosynthetic differences and root sugar levels of the 38 rice genotypes used in our study under the salinity condition.

### 3.3. Allantoin Is a Potential Metabolic Marker of Rice Tolerant to Growth Stress

Our metabolite profiling identified allantoin as a principal component increased by long-term exposure of mild salinity stress in rice roots. Allantoin is a catabolic intermediate of the purine ring and is known as a specific biomarker of oxidative stress in animal cells [32]. In plants, the purine catabolic pathway plays a major role in the remobilization of nitrogen resources between source and sink tissues [33]. Especially in nodulated tropical or subtropical legumes, ureides are the main nitrogen transport compounds [34]. In soybean, the ureides allantoin and allantoic acid are major products of nitrogen fixation in nodules and the dominant long-distance transport forms of nitrogen from root nodules to the shoot [35]. In addition to the function in nitrogen allocation, purine catabolites, such as the ureides allantoin and allantoate, are involved in protecting plants against abiotic stress [33]. For example, in Arabidopsis, regulation of purine catabolic compounds ureides has implications for optimal plant survival during nutrient remobilization and ROS scavenging [33,36]. In rice, allantoin is a phytochemical ubiquitously found in various tissues, and a high level of allantoin was found in rice root exudates, suggesting that allantoin may play a specialized function in rice [32]. There is a report that under the Zn deficiency and high bicarbonate stress condition, N-rich metabolites, including allantoin, are accumulated in rice roots [37]. In addition, the allantoin level was positively correlated with the drought tolerance traits of rice genotypes [38]. From our data, allantoin accumulation was observed in all 38 rice genotypes in response to salt stress. Therefore, allantoin is likely to be a distinctively conserved salt stress-responsive marker of rice roots. The function and source of this salt stress-accumulated N-rich metabolite allantoin in rice roots are unknown at present. A previous report suggests a protective function of allantoin under the stress condition in rice. For example, allantoin levels in rice grains and seedlings are positively correlated with the survival rate in seedbeds under cold and drought stress conditions [39]. In addition, exogenous application of allantoin increased plant biomass, soluble sugar and free proline content and decreased MDA content in rice seedlings, especially for allantoin-poor genotypes [39]. Taken together, these suggest that the endogenous allantoin level is likely to be positively related to growth potential in rice. In that sense, it is noteworthy that the fold increase of allantoin content by salt stress in the high growth and salt-tolerant group (HGST) was significantly higher (*p* < 0.05) than that in the low growth and salt-sensitive groups (LGSS) (Figure 4b).

In summary, our ^1^H-NMR analysis of salt-responsive metabolite markers in rice roots using 38 rice genotypes suggests a high energy demand of rice roots under long-term mild salt stress condition and emphasizes the important function of sucrose, glutamine and allantoin in salt-stressed rice roots. Adjustment of carbon and nitrogen metabolism in rice roots is likely to be closely related to maintain the growth potential and increase the stress tolerance of rice.

## 4. Experimental Section

### 4.1. Rice Growth and Salt Stress Treatment

Rice germplasms used in this study were obtained from the National Agrobiodiversity Center of Rural Development Administration (RDA-GenBank), Republic of Korea. The origin and accession numbers of the germplasms are shown in Table S1. Seeds were sterilized in 2% (*w*/*v*) sodium hypochlorite for 10 min, then washed with distilled water several times before sowing. Seeds were allowed to germinate in a plastic tray having 100 small squares, each with an area of 2 cm^2^. Four-day-old equally-sized seedlings were transferred to a hydroponic system with a half-strength Yoshida solution [40] in a growth chamber under the 13L:11D (light:dark) condition at 29 and 22 °C, respectively. After two weeks, to minimize the variation of growth and development among genotypes, a uniform size of plants with 3 or 4 foliar leaves were transferred to a half strength of Yoshida solution containing 43 mM NaCl and grown further for two weeks. Three replicates with 3 plants in each replicate were used. Within a replicate, the genotypes tested were randomly arranged to alleviate environmental and positioning effects. The Yoshida solution, either with or without 43 mM NaCl, was replaced every 3–4 days. At the end of the salinity treatment, whole seedlings were dried, and the biomass as total dry weight was measured for triplicates. Root samples for metabolite profiling were immediately harvested after salt treatment and stored at −80 °C.

### 4.2. Metabolite Extraction from Rice Roots

For metabolite analysis, the roots of nine plants with three biological repeats were pooled together and the metabolites were extracted. The metabolite extraction procedure was based on the methods reported by Choi *et al*. (2004) [41] with some modifications. Rice roots were ground in liquid nitrogen in a mortar with a pestle and freeze dried. Then, 60 mg of dried material were transferred to a 10-mL centrifuge tube to which 5 mL of 50% MeOH in water and 5 mL of chloroform were added. The mixture was vortexed for 30 s and sonicated for 1 min, then centrifuged at 3000 rpm for 20 min. The extraction procedure was performed twice. The upper and lower layer were collected separately and dried in a speed vacuum concentrator. The dried upper fractions were dissolved in 1 mL of KH_2_PO_4_ buffer in D_2_O.

### 4.3. NMR Measurements

Each water extract was solubilized in 1.0 mL D_2_O (KH_2_PO_4_ buffer, pH 6) and transferred into an NMR tube. ^1^H-NMR spectra were acquired on a Bruker DRX500 spectrometer equipped with a CPTXI probe operating at 500.13 MHz at 298 K. A noesypresat pulse sequence was applied to suppress the residual water signal. A total of 32 transients over a spectral region of 6009.6 Hz were collected, and the total number of data points was 16 K. The acquisition time was 1.4 s, with 1.5 s of water presaturation during the relaxation delay. The ^1^H-NMR spectra were assigned by comparing them with the Chenomx NMR suite 6.0 library and with data from the literature [42,43]. In addition, two-dimensional (2D) ^1^H–^1^H correlation spectroscopy (COSY) and total correlation spectroscopy (TOCSY) were used for further confirmation.

### 4.4. Data Analysis

A line broadening function of 0.3 Hz was applied to all ^1^H-NMR spectra before the Fourier transform (FT). Phase and baseline corrections were manually applied to all of the spectra using Chenomx NMR Suite 6.0 software (Chenomx Inc., Edmonton, AB, Canada). The NMR spectra were binned into 0.001 ppm segments between 0.2–9.0 ppm, and the region corresponding to water (4.67–5.17 ppm) was excluded. Each spectrum was normalized by setting the total spectral area to unity. The spectral data were converted to ASCII format and imported into MATLAB (Version 7, The MathWorks Inc., Natwick, MA, USA). The correlation optimized warping (COW) method was used to align all of the spectra [44]. Next, Simca-P+ (Version 12, Umetrics, Malmö, Sweden) was used to conduct multivariate statistical analyses of the data, and the analyses were performed using a mean-centered scaling of the variables from the binned data. Principal component analysis (PCA), which is an unsupervised pattern-recognition method, was applied to identify the intrinsic clusters and obvious outliers within the dataset. Partial least squares discriminant analysis (PLS-DA) and orthogonal partial least squares discriminant analysis (OPLS-DA) were also applied to maximize the separation between the groups.

The quality of the models is determined by the goodness of fit (R^2^Y) and predictability (Q^2^Y) values [45]. Furthermore, the PLS-DA model was validated by the permutation method, which evaluates whether the classification of the individuals into the two designed groups was significantly better than a random classification into two arbitrary groups. The peak intensities of several metabolites were integrated based on the chemical shift ranges, and relative integral intensities were obtained.

## Supplementary Materials

Supplementary materials can be found at http://www.mdpi.com/1422-0067/16/09/21959/s1.

## Abbreviations

Ala, alanine; CV, cultivar; GABA, γ-aminobutyric acid; Gln, glutamine; Glu, glutamate; GS, glutamine synthetase; HG, high growth; NMR, nuclear magnetic resonance; OPLS-DA, orthogonal partial least squares discriminant analysis; PCA, principal component analysis; PLS-DA, partial least squares discriminant analysis; LG, low growth; ST, salt tolerant; SS, salt sensitive; Thr, threonine; Val, valine; Ile, isoleucine.

## References

[1] Akbar M., Yabuno T., Nakao S. (1972). Breeding for saline-resistant varieties of rice. I. Variability for salt tolerance among some rice varieties. Jpn. J. Breed..

[2] Flowers T.J., Yeo A.R. (1981). Variability in the resistance of sodium chloride salinity within rice (*Oryza sativa* L.) varieties. New Phytol..

[3] Kahn M.S.A., Hamid A., Karim M.A. (1997). Effect of sodium chloride on germination and seedling characters of different types of rice (*Oryza sativa* L.). J. Agron. Crop Sci..

[4] Lutts S., Kinet J.M., Bouharmout J. (1995). Changes in plant response to NaCl during development of rice (*Oryza sativa* L.) varieties differing in salinity resistance. J. Exp. Bot..

[5] Yoshida S. (1981). Fundamentals of Rice Crop Science.

[6] Omokawa H., Aonuma S. (2002). Amelioration of the salt-stressed root growth of rice and normalization of the Na^+^ distribution between the shoot and root by (*S*)-α-methylbenzyl-2-fluoro-4-methylphenylurea. Biosci. Biotechnol. Biochem..

[7] Munns R., Tester M. (2008). Mechanisms of salinity tolerance. Annu. Rev. Plant Biol..

[8] Zhu J.K. (2001). Plant salt tolerance. Trends Plant Sci..

[9] Ji H., Pardo J.M., Batelli G., van Oosten M.J., Bressan R.A., Li X. (2013). The salt overly sensitive (SOS) pathway: Established and emerging roles. Mol. Plant.

[10] Martínez-Atienza J., Jiang X., Garciadeblas B., Mendoza I., Zhu J.K., Pardo J.M., Quintero F.J. (2007). Conservation of the salt overly sensitive pathway in rice. Plant Physiol..

[11] Kim H., Lee K., Hwang H., Bhatnagar N., Kim D.Y., Yoon I.S., Byun M.O., Kim S.T., Jung K.H., Kim B.G. (2014). Overexpression of PYL5 in rice enhances drought tolerance, inhibits growth, and modulates gene expression. J. Exp. Bot..

[12] Liu C., Mao B., Ou S., Wang W., Liu L., Wu Y., Chu C., Wang X. (2014). OsbZIP71, a bZIP transcription factor, confers salinity and drought tolerance in rice. Plant Mol. Biol..

[13] Diédhiou C.J., Popova O.V., Dietz K.J., Golldack D. (2008). The SNF1-type serine-threonine protein kinase SAPK4 regulates stress-responsive gene expression in rice. BMC Plant Biol..

[14] Fiehn O. (2002). Metabolomics: The link between genotypes and phenotype*s*. Plant Mol. Biol..

[15] Kim J.K., Bamba T., Harada K., Fukusaki E., Kobayashi A. (2007). Time-course metabolic profiling in *Arabidopsis thaliana* cell cultures after salt stress treatment. J. Exp. Bot..

[16] Kim H.K., Choi Y.H., Verpoorte R. (2010). NMR-based metabolomic analysis of plants. Nat. Protoc..

[17] Ali K., Iqbal M., Fortes A.M., Pais M.S., Korthout H.A., Verpoorte R., Choi Y.H. (2013). Red wines attenuate TNFα production in human histiocytic lymphoma cell line: An NMR spectroscopy and chemometrics based study. Food Chem..

[18] Wang Z., Chen Z., Yang S., Wang Y., Yu L., Zhang B., Rao Z., Gao J., Tu S. (2012). ^1^H-NMR-based metabolomic analysis for identifying serum biomarkers to evaluate methotrexate treatment in patients with early rheumatoid arthritis. Exp. Ther. Med..

[19] Teixeira J., Fidalgo F. (2009). Salt stress affects glutamine synthetase activity and mRNA accumulation on potato plants in an organ-dependent manner. Plant Physiol. Biochem..

[20] Wang H., Zhang M., Guo R., Shi D., Liu B., Lin X., Yang C. (2012). Effects of salt stress on ion balance and nitrogen metabolism of old and young leaves in rice (*Oryza sativa* L.). BMC Plant Biol..

[21] Funayama K., Kojima S., Tabuchi-Kobayashi M., Sawa Y., Nakayama Y., Hayakawa T., Yamaya T. (2013). Cytosolic glutamine synthetase 1;2 is responsible for the primary assimilation of ammonium in rice roots. Plant Cell Physiol..

[22] Kusano M., Tabuchi M., Fukushima A., Funayama K., Diaz C., Kobayashi M., Hayashi N., Tsuchiya Y.N., Takahashi H., Kamata A. (2011). Metabolomics data reveal a crucial role of cytosolic glutamine synthetase 1;1 in coordinating metabolic balance in rice. Plant J..

[23] Siahpoosh M.R., Sanchez D.H., Schlereth A., Scofield G.N., Furbank R.T., van Dongen J.T., Kopka J. (2012). Modification of *OsSUT1* gene expression modulates the salt response of rice *Oryza sativa* cv. Taipei 309. Plant Sci..

[24] Cai H., Zhou Y., Xiao J., Li X., Zhang Q., Lian X. (2009). Overexpressed glutamine synthetase gene modifies nitrogen metabolism and abiotic stress responses in rice. Plant Cell Rep..

[25] Hirano T., Satoh Y., Ohki A., Takada R., Arai T., Michiyama H. (2008). Inhibition of ammonium assimilation restores elongation of seminal rice roots repressed by high levels of exogenous ammonium. Physiol. Plant..

[26] Rosa M., Prado C., Podazza G., Interdonato R., González J.A., Hilal M., Prado F.E. (2009). Soluble sugars—Metabolism, sensing and abiotic stress: A complex network in the life of plants. Plant Signal. Behav..

[27] Sanchez D.H., Siahpoosh M.R., Roessner U., Udvardi M., Kopka J. (2008). Plant metabolomics reveals conserved and divergent metabolic responses to salinity. Physiol. Plant..

[28] Liu D., Ford K.L., Roessner U., Natera S., Cassin A.M., Patterson J.H., Bacic A. (2013). Rice suspension cultured cells are evaluated as a model system to study salt responsive networks in plants using a combined proteomic and metabolomic profiling approach. Proteomics.

[29] Lemoine R., la Camera S., Atanassova R., Dédaldéchamp F., Allario T., Pourtau N., Bonnemain J.L., Laloi M., Coutos-Thévenot P., Maurousset L. (2013). Source-to-sink transport of sugar and regulation by environmental factors. Front. Plant Sci..

[30] Kanemura T., Homma K., Ohsumi A., Shiraiwa T., Horie T. (2007). Evaluation of genotypic variation in leaf photosynthetic rate and its associated factors by using rice diversity research set of germplasm. Photosynth. Res..

[31] Boriboonkaset T., Theerawitaya C., Yamada N., Pichakum A., Supaibulwatana K., Cha-Um S., Takabe T., Kirdmanee C. (2013). Regulation of some carbohydrate metabolism-related genes, starch and soluble sugar contents, photosynthetic activities and yield attributes of two contrasting rice genotypes subjected to salt stress. Protoplasma.

[32] Chung W.Y., Benzie I.F. (2013). Plasma allantoin measurement by isocratic liquid chromatography with tandem mass spectrometry: Method evaluation and application in oxidative stress biomonitoring. Clin. Chim. Acta.

[33] Werner A.K., Witte C.P. (2011). The biochemistry of nitrogen mobilization: Purine ring catabolism. Trends Plant Sci..

[34] Tegeder M. (2014). Transporters involved in source to sink partitioning of amino acids and ureides: Opportunities for crop improvement. J. Exp. Bot..

[35] Collier R., Tegeder M. (2012). Soybean ureide transporters play a critical role in nodule development, function and nitrogen export. Plant J..

[36] Brychkova G., Alikulov Z., Fluhr R., Sagi M. (2008). A critical role for ureides in dark and senescence-induced purine remobilization is unmasked in the *Atxdh1* Arabidopsis mutant. Plant J..

[37] Wang P., Kong C.H., Sun B., Xu X.H. (2012). Distribution and function of allantoin (5-ureidohydantoin) in rice grains. J. Agric. Food Chem..

[38] Rose M.T., Rose T.J., Pariasca-Tanaka J., Yoshihashi T., Neuweger H., Goesmann A., Frei M., Wissuwa M. (2012). Root metabolic response of rice (*Oryza sativa* L.) genotypes with contrasting tolerance to zinc deficiency and bicarbonate excess. Planta.

[39] Degenkolbe T., Do P.T., Kopka J., Zuther E., Hincha D.K., Köhl K.I. (2013). Identification of drought tolerance markers in a diverse population of rice cultivars by expression and metabolite profiling. PLoS ONE.

[40] Yoshida S., Forno D., Cook J., Gomez K. (1976). Laboratory Manual for Physiological Studies of Rice.

[41] Choi H.K., Choi Y.H., Verberne M., Lefeber A.W., Erkelens C., Verpoorte R. (2004). Metabolic fingerprinting of wild type and transgenic tobacco plants by ^1^H-NMR and multivariate analysis technique. Phytochemistry.

[42] Baker J.M., Hawkins N.D., Ward J.L., Lovegrove A., Napier J.A., Shewry P.R., Beale M.H. (2006). A metabolomic study of substantial equivalence of field-grown genetically modified wheat. Plant Biotechnol. J..

[43] Fan T.M.T. (1996). Metabolite profiling by one- and two-dimensional NMR analysis of complex mixtures. Prog. Nucl. Magn. Reson. Spectrosc..

[44] Larsen F.H., van den Berg F., Engelsen S.B. (2006). An exploratory chemometric study of ^1^H-NMR spectra of table wine. J. Chemom..

[45] Eriksson L., Johansson E., Kettaneh-Wold N., Trygg J., Wikstrom C., Wold S. (2006). Multi and Megavariate Data Analysis Part I: Basic Principles and Applications.

